# Author Correction: Development of a functional salivary gland tissue chip with potential for high-content drug screening

**DOI:** 10.1038/s42003-021-02103-3

**Published:** 2021-04-30

**Authors:** Yuanhui Song, Hitoshi Uchida, Azmeer Sharipol, Lindsay Piraino, Jared A. Mereness, Matthew H. Ingalls, Jonathan Rebhahn, Shawn D. Newlands, Lisa A. DeLouise, Catherine E. Ovitt, Danielle S. W. Benoit

**Affiliations:** 1 Department of Biomedical Engineering, University of Rochester, Rochester, NY USA; 2 Center for Oral Biology, University of Rochester Medical Center, Rochester, NY USA; 3 Department of Biomedical Genetics, University of Rochester Medical Center, Rochester, NY USA; 4 Department of Dermatology, University of Rochester Medical Center, Rochester, NY USA; 5 Department of Environmental Medicine, University of Rochester Medical Center, Rochester, NY USA; 6 David H. Smith Center for Vaccine Biology and Immunology, University of Rochester Medical Center, Rochester, NY USA; 7 Department of Otolaryngology, University of Rochester Medical Center, Rochester, NY USA; 8 Wilmot Cancer Institute, University of Rochester Medical Center, Rochester, NY USA; 9 Department of Neuroscience, University of Rochester Medical Center, Rochester, NY USA; 10 Materials Science Program, University of Rochester, Rochester, NY USA; 11 Department of Chemical Engineering, University of Rochester, Rochester, NY USA; 12 Center for Musculoskeletal Research, University of Rochester Medical Center, Rochester, NY USA

**Keywords:** High-throughput screening, Assay systems, Biomimetic synthesis

Correction to: *Communications Biology* 10.1038/s42003-021-01876-x, published online 19 March 2021.

In the original version of the Article, the Acknowledgments listed the award numbers UG3DE027695 and UH3 DE002212. Those award numbers should have been UH3 DE027695 and UG DE027695, respectively.

This has now been corrected in the PDF and HTML versions of the Article.

