# Author Correction: Development of a functional salivary gland tissue chip with potential for high-content drug screening

**DOI:** 10.1038/s42003-022-03274-3

**Published:** 2022-03-30

**Authors:** Yuanhui Song, Hitoshi Uchida, Azmeer Sharipol, Lindsay Piraino, Jared A. Mereness, Matthew H. Ingalls, Jonathan Rebhahn, Shawn D. Newlands, Lisa A. DeLouise, Catherine E. Ovitt, Danielle S. W. Benoit

**Affiliations:** 1 Department of Biomedical Engineering, University of Rochester, Rochester, NY USA; 2 Center for Oral Biology, University of Rochester Medical Center, Rochester, NY USA; 3 Department of Biomedical Genetics, University of Rochester Medical Center, Rochester, NY USA; 4 Department of Dermatology, University of Rochester Medical Center, Rochester, NY USA; 5 Department of Environmental Medicine, University of Rochester Medical Center, Rochester, NY USA; 6 David H. Smith Center for Vaccine Biology and Immunology, University of Rochester Medical Center, Rochester, NY USA; 7 Department of Otolaryngology, University of Rochester Medical Center, Rochester, NY USA; 8 Wilmot Cancer Institute, University of Rochester Medical Center, Rochester, NY USA; 9 Department of Neuroscience, University of Rochester Medical Center, Rochester, NY USA; 10 Materials Science Program, University of Rochester, Rochester, NY USA; 11 Department of Chemical Engineering, University of Rochester, Rochester, NY USA; 12 Center for Musculoskeletal Research, University of Rochester Medical Center, Rochester, NY USA

**Keywords:** High-throughput screening, Assay systems, Biomimetic synthesis

Correction to: *Communications Biology* 10.1038/s42003-021-01876-x, published online 19 March 2021.

The Supplementary Information file of the original version of the Article omitted Supplementary Tables 1 and 2. This has now been corrected in the Supplementary Information file.

## Supplementary information

Updated Supplementary Information

