# Supplementary material for: Development of a functional salivary gland tissue chip with potential for high-content drug screening
Source: Commun Biol. 2021 Mar 19;4:361. doi: 10.1038/s42003-021-01876-x (PMC7979686; doi:10.1038/s42003-021-01876-x)
Supplement: Supplementary file 2 — Supplementary Information [file 42003_2021_1876_MOESM2_ESM.pdf]

Supplemental Figures

Supplementary Figure 1: Isolation and viability of AIDUC for mouse salivary gland tissue chip.

Supplementary Figure 2: AIDUC characterization by IHC staining with acinar and duct cell-specific markers.

Supplementary Figure 3: Microbubble (MB) array fabrication steps.

Supplementary Figure 4: Mouse AIDUCs seeded in MB-hydrogels lack detectable expression of the neuronal marker, ***β***III-tubulin (*Tubb3*).

Supplementary Figure 5: Characterization of SGm by IHC staining with acinar and duct cell-specific markers.

Supplementary Figure 6: SGm cultured in MB-hydrogel promotes formation of tight junctions and apicobasal polarization.

Supplementary Figure 7: Characterization of SGm using Periodic acid-Schiff's – Alcian Blue (PAS-AB) staining.

Supplementary Figure 8: Cytokeratin-positive cell outgrowth from MB is observed after day 7 in SGm.

Supplementary Figure 9: SGm in MB-hydrogels express the ***β***-adrenergic receptor and are responsive to stimulation with isoproterenol (IPN).

Supplementary Figure 10: Human SGm raw fluorescence intensity to enable normalized amylase measurements.

Supplementary Figure 11: Timeline of SGm irradiation, radioprotection, and characterization.

Supplementary Table 1: Mouse and human primers for qPCR.

Supplementary Table 2: Antibody Information for IHC.

Supplementary Videos

Supplementary Video 1: Timelapse of formation of SGm in MB-hydrogel over 4 days.

Supplementary Video 2: Representative calcium imaging of mouse SGm at Day 7 stimulated with CCh.

Supplementary Video 3: Representative of whole-chip calcium imaging on mouse SGm at Day 7 stimulated with CCh.

Supplementary Video 4: Representative calcium imaging of human SGm at Day 7 stimulated with CCh.

Supplementary Video 5: Representative whole-chip calcium imaging on human SGm at Day 7 stimulated with CCh.

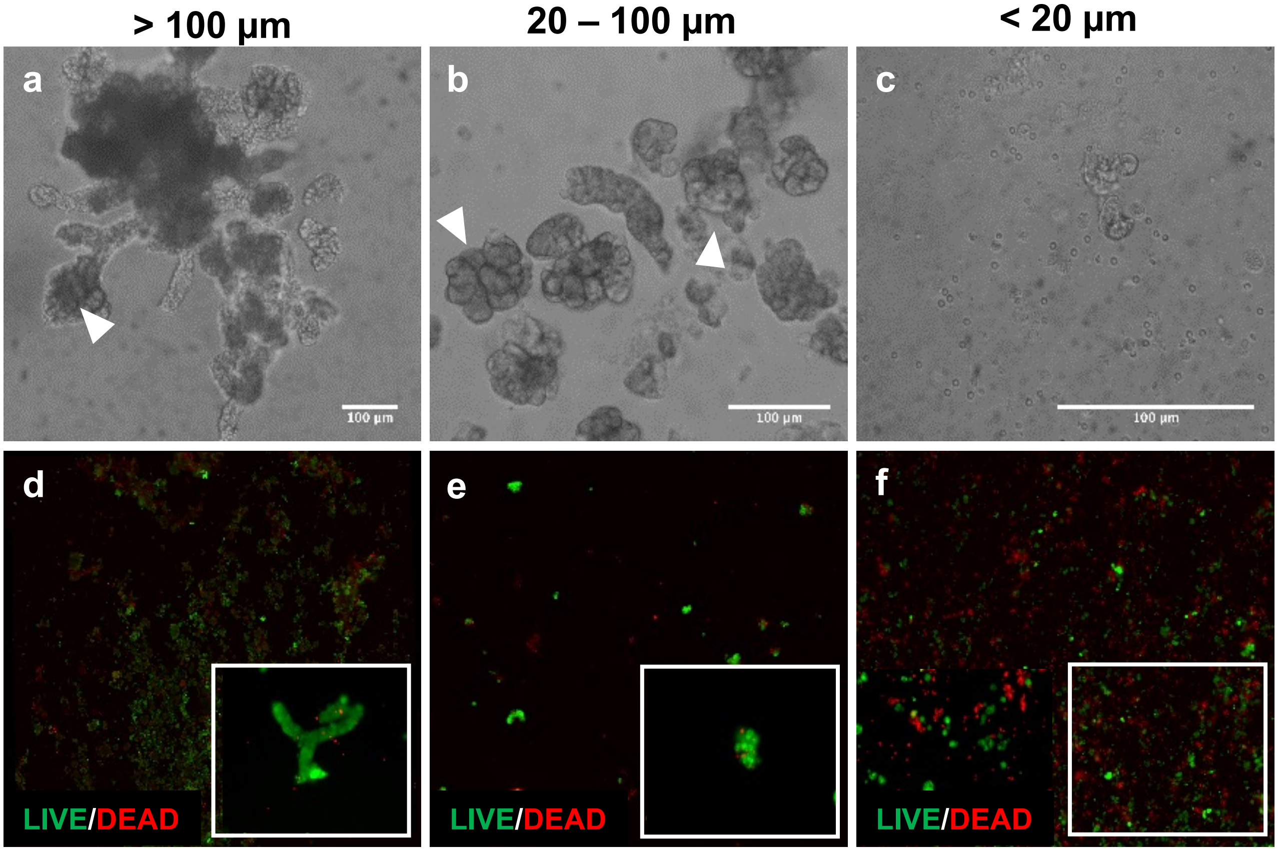

Supplementary Figure 1: Isolation and viability of AIDUC for salivary gland tissue chip. **a-c**, Representative brightfield and **d-f**, fluorescent LIVE/DEAD stained images of mouse submandibular gland digest fractions of a & d, >100 µm, b & e, 20 – 100 µm, and c & f, <20 µm in size. Scale bars = 100 µm.

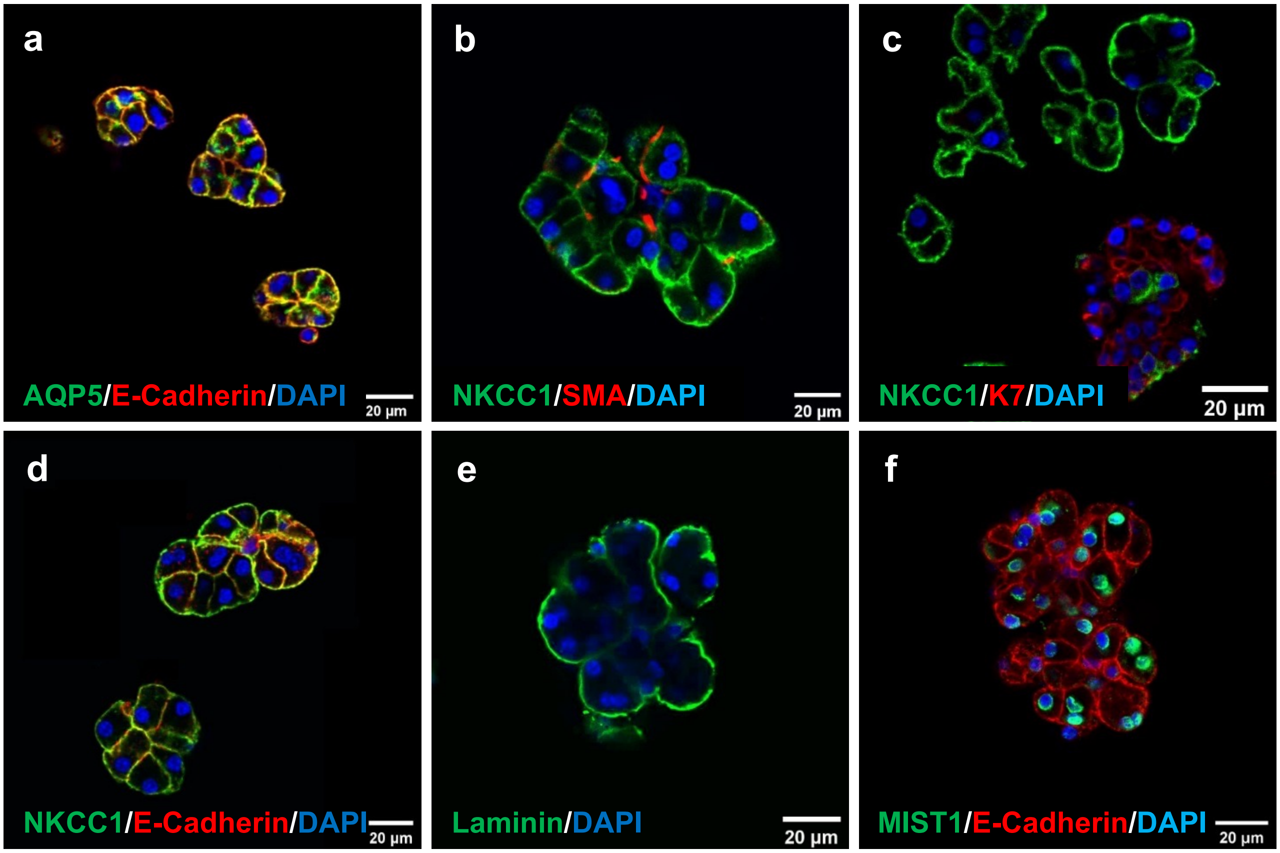

Supplementary Figure 2: AIDUC characterization using IHC staining with acinar and duct cell-specific markers. Representative immunofluorescent images of freshly isolated AIDUCs stained with acinar cell markers, AQP5 (**a,** green), NKCC1 (**b**, **c**, **d**, green) and MIST1 (**f**, green), duct cell marker, K7 (**c**, red), myoepithelial cell marker, SMA (**b**, red), epithelial cell marker, E-Cadherin (**a**, **d**, **f**, red) and basement membrane protein, laminin (**e**, green). Nuclei are stained with DAPI (**a** – **f**, blue). Scale bars = 20 µm.

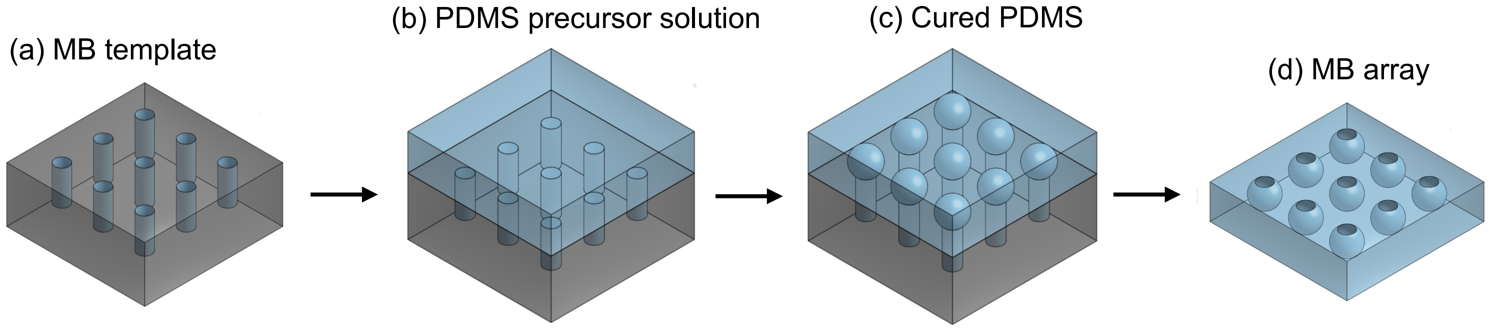

Supplemental Figure 3: Microbubble (MB) array fabrication steps. (a) A template (gray) with deep pits is used for MB fabrication using gas expansion molding[41,42]. (b) PDMS precursor solution (blue) is poured over the template. (c) The PDMS solution is cured at 100°C for 2 hours. Air from the pits in the template nucleate microbubble formation in the PDMS. (d) The cured MB array is peeled off the template, flipped over, and glued into the bottom of well plates. Note: not drawn to scale. 3D rendering was done using Onshape. MB parts are commercially available at Nidus MB Technologies (www.nidusmbt.com).

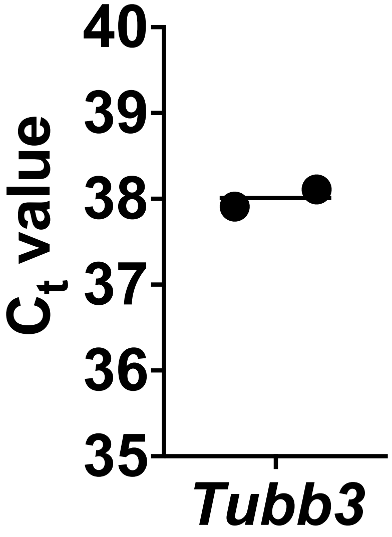

Supplementary Figure 4: Mouse AIDUCs at day 0 lack detectable expression of the neuronal marker, *β*III-tubulin (*Tubb3*). Data are mean ± standard deviation from N=6 mice, n=6 samples/mouse where each marker is an average Ct value. Note: 4/6 AIDUCs isolated exhibited undetectable Tubb3 gene expression, hence only 2 data points are included herein.

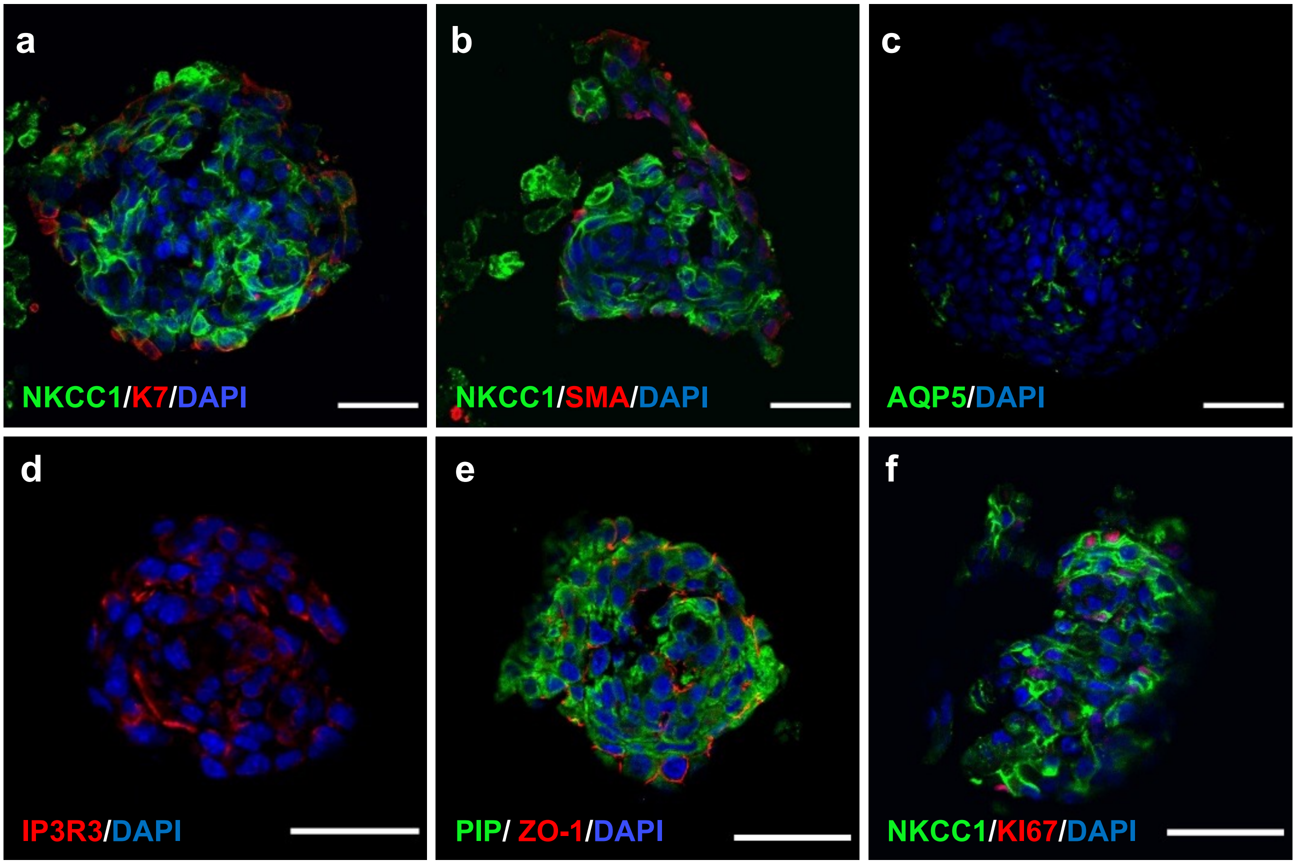

Supplementary Figure 5: Characterization of SGm using IHC staining with acinar and duct cell-specific markers. Representative immunofluorescent images of day 7 SGm stained for acinar cell markers, NKCC1 (**a**, **b**, **f**, green), AQP5 (**c**, green), and IP3R3 (**d**, red), duct cell marker, K7 (**a**, red), myoepithelial cell marker, SMA (**b**, red), tight junction protein, ZO-1 (**e**, red), and proliferation marker, Ki67 (**f**, red). Nuclei are stained with DAPI (**a** – **f**, blue). Scale bars = 40 µm.

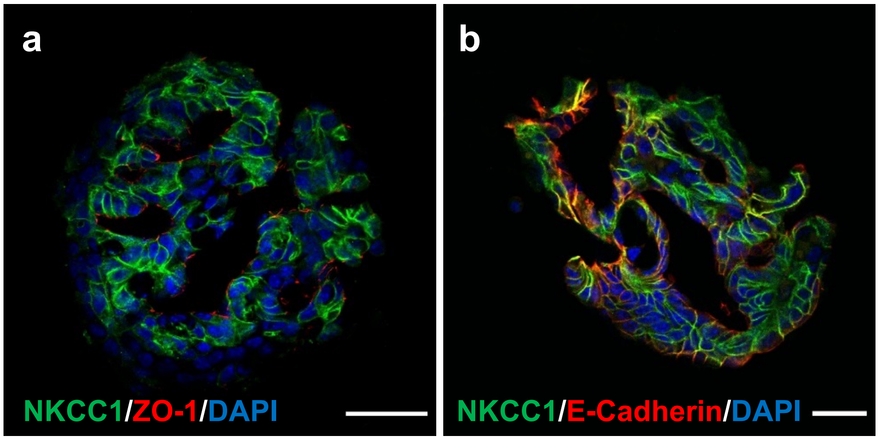

Supplementary Figure 6: SGm cultured in MB-hydrogel promotes formation of tight junctions and apicobasal polarization. Representative images of IHC staining of SGm at day 14 of culture. Shown are the acinar cell marker, NKCC1 (**a**, **b**, green), tight junction protein, ZO-1 (**a**, red), and the epithelial cell marker, E-Cadherin (**b**, red). Nuclei are stained with DAPI (**a**, **b** blue). Scale bars = 40 μm.

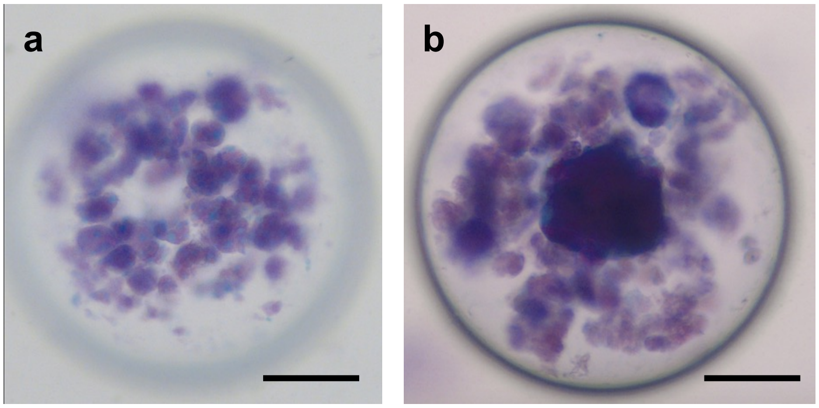

Supplementary Figure 7: Characterization of SGm using Periodic acid-Schiff's – Alcian Blue (PAS-AB) staining. Staining of day 0 (a) and day 7 (b) SGm in the MB chip showing presence of both acidic and neutral mucins. Scale bar = 100 µm.

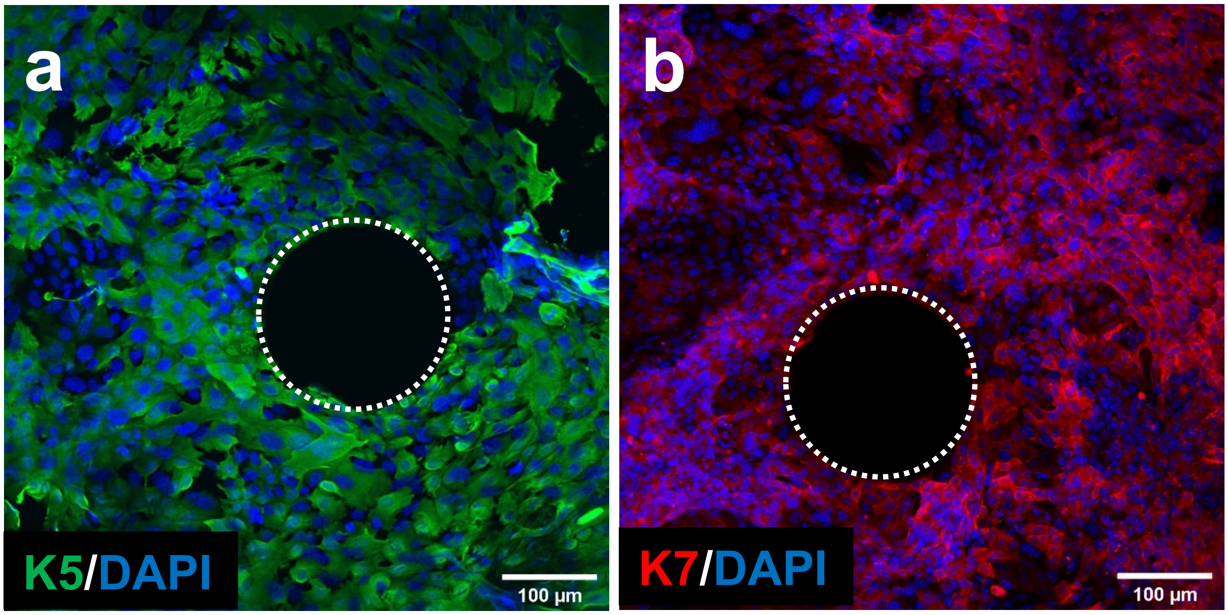

Supplementary Figure 8: Cytokeratin-positive cell outgrowth from MB is observed after day 7 in SGm. Representative immunofluorescent images of day 14 SGm culture focused on MB array chip surface stained for cytokeratin 5 (K5) (**a**, green) and cytokeratin 7 (K7) (**b**, red). Nuclei are stained with DAPI (blue). White circles indicate the opening of the MBs.

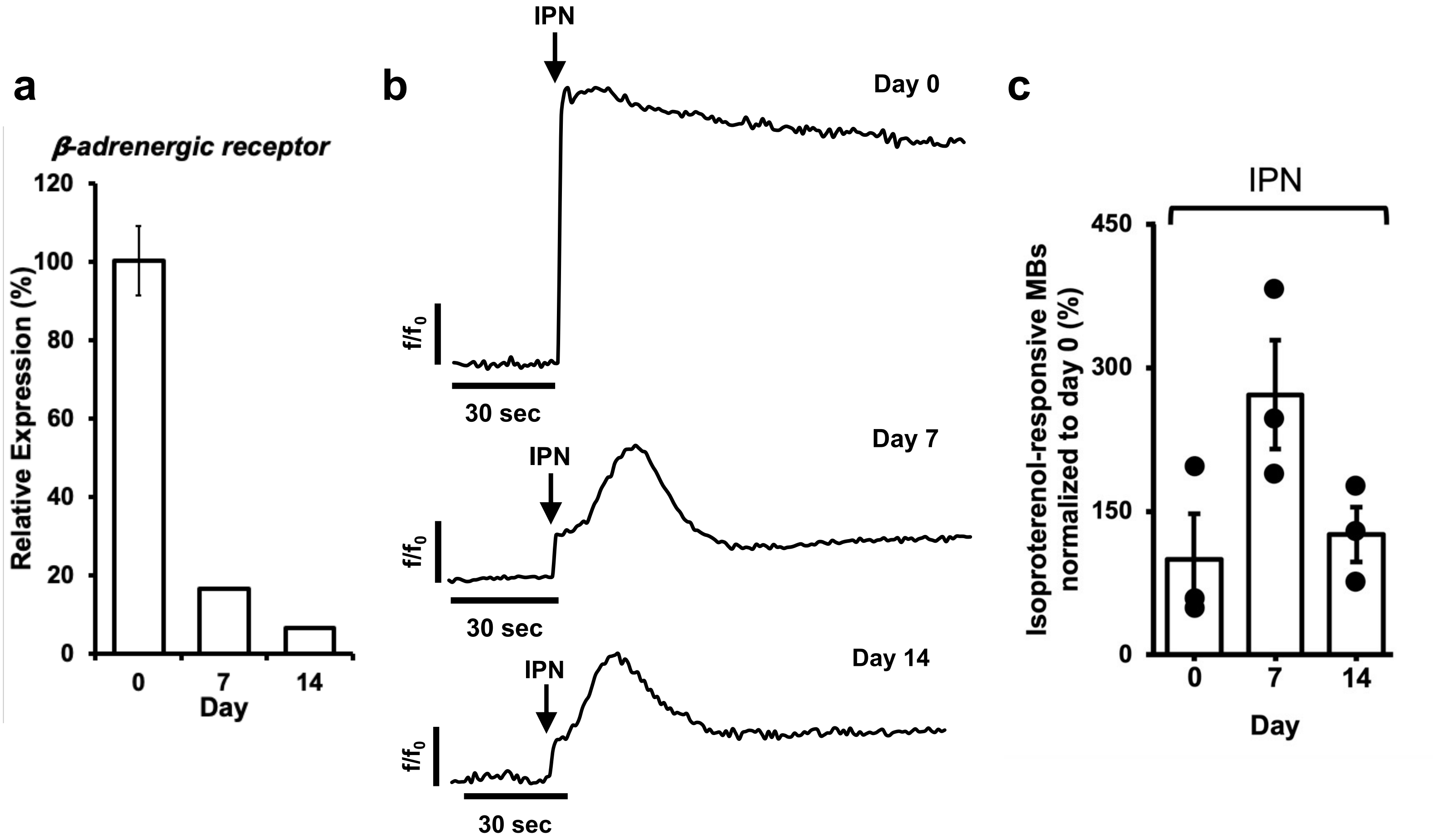

Supplementary Figure 9: Mouse SGm in MB-hydrogels express the *β*-adrenergic receptor (ADRA2 receptor) and are responsive to stimulation with adrenergic agonist isoproterenol (IPN). *a*, Gene expression of the ***β***-adrenergic receptor. ***b***, Representative fluorescent traces of responsive SGm at day 0, 7, and 14 upon ISN stimulation. Data are represented as fluorescent intensity (f) divided by fluorescent at time 0 (f_0). m, Percent of SGm in MBs responsive to IPN. Response is characterized by a significant difference between baseline timepoints and agonist timepoints via unpaired t-tests corrected for multiple comparisons using Holm-Sidak with α= 0.05. Data is graphed and normalized to day 0 as 100%. Gene expression data are mean ± standard deviation from N=1, n=3 per timepoint. Fluorescent calcium flux data are mean ± SEM, N = 3, n = 112 – 272 per timepoint.

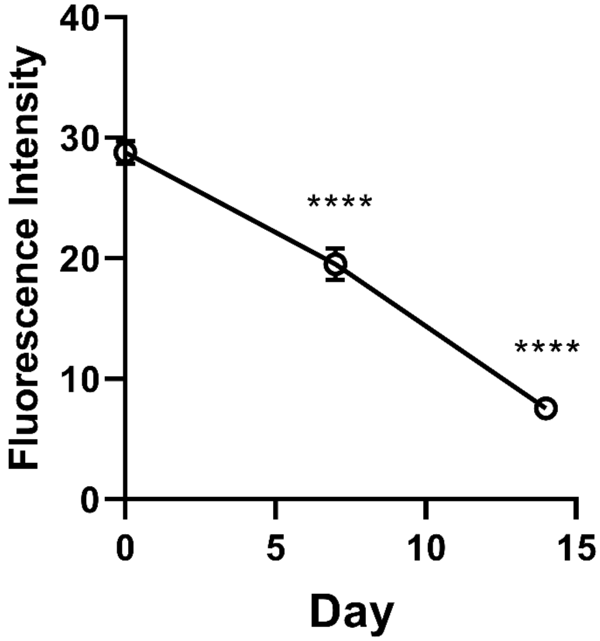

Supplementary Figure 10: Human SGm raw fluorescence intensity to enable normalized amylase measurements. Quantification of fluorescence intensity generated by amylase activity in the MB-hydrogel at days 0, 7, and 14, represented as the mean and standard deviation of the fluorescence intensity from individual MBs. ****$p < 0.0001$

Supplementary Figure 11: Timeline of SGm irradiation, radioprotection, and characterization. Timeline depicting seeding, WR1065 dosing and irradiation schedule, and timepoints for analyses used in singular and fractionated irradiation experiments.

Singular dose irradiation:

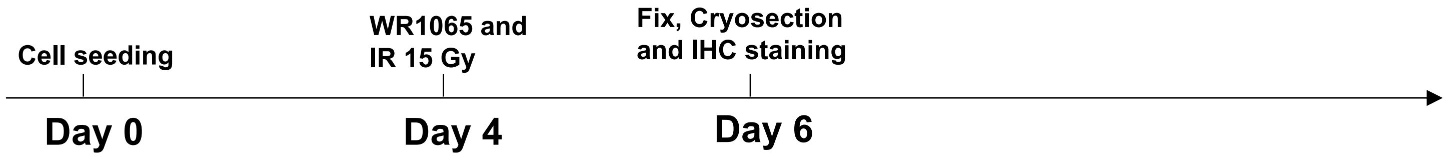

Fractionated irradiation:

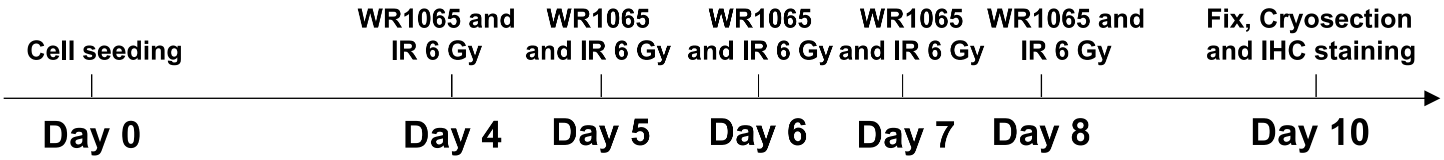

Supplementary Table 1: Mouse and human primers for PCR

Mouse		Sequence	Ref.
Rps29	F	AATACGGGCTGAACATGTGC	PrimerBLAST design with MIQE validation
	R	AGCATGATCGGTTCCACTTG	
Mist1	F	GCTGACCGCCACCATACTTAC	Shubin et al. Acta Biomaterialia 2017
	R	TGTGTAGAGTAGCGTTGCAGG	
Pip	F	GGGTCTCTCATTCACATTCAGTG	Shubin et al. Acta Biomaterialia 2017
	R	TGATCTCCTGATTTTCCTGTGCT	
Nkcc1	F	TTCCGCGTGAACTTCGTGG	Shubin et al. Acta Biomaterialia 2017
	R	TTGGTGTGGGTGTCATAGTAGT	
Aqp5	F	GTGAGTGGTGGCCACATCAATCC	Shubin et al. Acta Biomaterialia 2017
	R	GGGAGTCCGTGGAGGAGAAGAT	
M3r	F	GGGGAACTTAGCCTGTGACC	PrimerBLAST design with MIQE validation
	R	GTTGTTCGTTTGGCTCGG	
P2y2	F	CGCTTCAACGAGGACTTCA	PrimerBLAST design with MIQE validation
	R	GGTTTTGAGGCGGCATAGGA	
P2x7	F	GCTGCTTGGGAAAAGTCTG	PrimerBLAST design with MIQE validation
	R	TTTCCACACTGGCACCAAC	
Krt5	F	TCCTGTTGAACGCCGCTGAC	Emmerson et al. eLIFE 2017
	R	CGGAAGGACACACTGGACTGG	
Krt7	F	CAGGCAGAGATTGACACCTT	Yamaguchi et al. Development 2006
	R	GCGCCAGCTTGGTGTTCAG	
α-Sma	F	GTCCCAGACATCAGGGAGTAA	Wu et al. J Immunol 2014
	R	TCGGATACTTCAGCGTCAGGA	
Amy1	F	ACTGGGCTTTGTCAGAAACT	Yamagishi et al. Acta Histchem Cytochem 2014
	R	GGGTCTTCGGCAGAGTTACT	
Cst3	F	AGGAGGCAGATGCCAATGAG	Spandidos et al. Nucl Acids Res 2010
	R	GGGCTGGTCATGGAAAGGA	PrimerBank ID 31981822a1
Cst10	F	CTGGCTGTGATCCCTGAAGC	Spandidos et al. Nucl Acids Res 2010
	R	ACTTTCTGCACCTCCTTATCCT	PrimerBank ID 10946758a1
Lyz2	F	ATGGAATGGCTGGCTACTATGG	Spandidos et al. Nucl Acids Res 2010
	R	ACCAGTATCGGCTATTGATCTGA	PrimerBank ID 8393739a1
Muc5b	F	GCACGTAAATGCGACTGTCT	Tetaert et al. Respiratory Research 2007
	R	ATGGACCTTGCTCTCCTGAC	
Ngf	F	CCAGTGAAATTAGGCTCCCTG	Spandidos et al. Nucl Acids Res 2010
	R	CCTTGGCAAAACCTTTATTGGG	PrimerBank ID 7305313a1
Smr3a	F	GGCCCTAGAAGACATGATCCT	Spandidos et al. Nucl Acids Res 2010
	R	GGAGACGGATTGCTTGGAGG	PrimerBank ID 14389425a1
β_1-adrenergic receptor	F	GGAGCTCCCTCGGACGAC	Mu et al. Nature Med 2012
	R	AGCCTGGCTCTCTACACCTTG	
βIII-tubulin	F	GGCAACTATGTAGGGGACTCAG	Pasquarella et al. Development 2016
	R	ATGGTTCCAGGTTCCAAGTC	

Human		Sequence	Ref.
RPS29	F	GCACTGCTGAGAGCAAGATG	de Jonge et al. PLOS ONE 2007
	R	ATAGGCAGTGCCAAGGAAGA	
MIST1	F	CGGATGCACAAGCTAAATAACG	Emmerson et al. eLIFE 2017
	R	GCCGTCAGCGATTTGATGTAG	
NKCC1	F	TTCCGCGTGAACTTCGTGG	Farmer et al. Development 2017
	R	TTGGTGTGGGTGTCATAGTAG	
AQP5	F	CTGTCCATTGGCCTGTCTGTC	Farmer et al. Development 2017
	R	GGCTCATACGTGCCTTTGATC	
M3R	F	TCACAGCACCATCCTCAAC	PrimerBLAST design with MIQE validation
	R	GCTTGTCGGCTTTCCTCTC	
P2Y2	F	ACCCTCAACGCCATCAAC	PrimerBLAST design with MIQE validation
	R	GCCCAGCCAGGAAGTAGAG	
P2X7	F	GACGCTCTGTTCCTCTGACC	Zhang et al. Adv Med Sci 2019
	R	CACCAGGCAGAGACTTCACA	

Supplementary Table 2: Antibody Information for IHC

Antigen	Company	Cat.#	Species
Ki67	BD pharmingen	550609	Mouse
AQP5	Abcam	ab92320	Rabbit
IP3R3	BD Transduction Laboratories™	610312	Mouse
NKCC1(D208R)	Cell Signaling	85403	Rabbit
K7	Abcam	ab9021	Mouse
SMA	Thermo Scientific™	MS-113-p	Mouse
PIP	Generous gift from Dr. Yvonne Myal		Rabbit
Amylase	Cell Signaling	3796	Rabbit
53BP1	Abcam	ab175933	Rabbit
γH2AX	Millipore	05-636-I	Mouse
ZO-1	Thermo Scientific™	339100	Mouse
E-Cadherin	BD Transduction Laboratories™	610181	Mouse
Laminin	Thermo Scientific™	PA1-16730	Rabbit
Mist 1	Abcam	ab187978	Rabbit
Donkey anti-Rabbit IgG (H+L) Highly Cross-Adsorbed Secondary Antibody, Alexa Fluor 488	Thermo Scientific™	A21206	Donkey
Donkey anti-Mouse IgG (H+L) Highly Cross-Adsorbed Secondary Antibody, Alexa Fluor 594	Thermo Scientific™	A21203	Donkey
