# Supplementary material for: Development of a functional salivary gland tissue chip with potential for high-content drug screening
Source: Commun Biol. 2021 Mar 19;4:361. doi: 10.1038/s42003-021-01876-x (PMC7979686; doi:10.1038/s42003-021-01876-x)
Supplement: Supplementary file 3 — Description of Additional Supplementary Files [file 42003_2021_1876_MOESM3_ESM.pdf]

Description of Additional Supplementary Files

File Name: Supplementary Movie 1

Description: Timelapse of formation of SGm in MB-hydrogel over 4 days.

File Name: Supplementary Movie 2

Description: Representative calcium imaging of mouse SGm at Day 7 stimulated with CCh.

File Name: Supplementary Movie 3

Description: Representative of whole-chip calcium imaging on mouse SGm at Day 7 stimulated with CCh.

File Name: Supplementary Movie 4

Description: Representative calcium imaging of human SGm at Day 7 stimulated with CCh.

File Name: Supplementary Movie 5

Description: Representative whole-chip calcium imaging on human SGm at Day 7 stimulated with CCh.

File name: Supplementary Data 1

Description: Source data for the main and supplementary figures.
